# Editorial: Application of Plant Natural Products and New Emerging Technologies for the Postharvest Storage of Fruits

**DOI:** 10.3389/fnut.2022.884438

**Published:** 2022-04-07

**Authors:** Chunpeng Wan, Kannan R. R. Rengasamy

**Affiliations:** ^1^Jiangxi Key Laboratory for Postharvest Technology and Nondestructive Testing of Fruits and Vegetables, College of Agronomy, Jiangxi Agricultural University, Nanchang, China; ^2^Indigenous Knowledge Systems Centre, Faculty of Natural and Agricultural Sciences, North-West University, Mmabatho, South Africa

**Keywords:** plant natural products, postharvest storage, essential oils, fruit quality, fruit safety

Postharvest storage of fruits had a significant role in improving the shelf-life and providing food for a longer time, thus mitigating food security and food losses, especially perishable fruits. Postharvest loss is the most significant loss in the food supply chain, which needs utmost importance to ensure food security for the future (1). The major postharvest losses are mechanical distortion and microbial degradation that significantly reduced the quality and quantity of the fresh fruits. Traditionally, fruits were dried in the sun and stored in a closed container, thus removing the moisture and avoiding air contact. With modernization, chemicals (pesticides, fungicides) and storage technologies (mainly cold storage) have been used for many years. The escalated public awareness about the use of chemicals in postharvest quality maintenance of fruits has enabled more attention toward plant-based natural compounds and emerging green technologies. These emerging technologies mainly include (smart packaging, plasma activated water, electrolyzed water, etc.). A lot of research has been done on these technologies for providing more safer and greener food products (2, 3). The revolution in preservation and storage technologies provides new solutions, including new cooling systems (with atmospheric gaseous and ethylene control), smart packaging, edible film/coating packaging, nanotechnologies, etc. (4). However, in most cases, the use of synthetic pesticides and fungicides is significant, which leads to lethal human health and environmental effects. Hence, the search for alternative solutions is in demand. Natural compounds derived from plants have been ranked superior in controlling postharvest losses. Plant natural products are environmentally friendly, safe for health, and prevent postharvest losses. Various materials developed from natural plant products have been applied to maintain postharvest fruit quality. In line with this, the present special issues would like to cover the advances in using natural products in postharvest storage of fruits and vegetables.

Essential oils have been known for their antimicrobial and aromatic nature. Several plants bearing fruits contain essential oils (5). In a published review on the biocontrol potential of essential oils in organic horticulture systems (Chang et al.), it has been postulated that essential oil and its products are potentially promising candidates as biocontrol agents due to their safe, bioactive, biodegradable, ecologically, and economically viable properties. This review highlighted some important plants for essential oil extraction, such as clove, ginger, oregano, rosemary, sage, and thyme. Also, advanced techniques such as solvent-free microwave extraction, ultrasonic-microwave assisted extraction, supercritical fluid extraction, subcritical water extraction, and ohmic heating-assisted extraction were discussed, which affect the oil quality and yield. The biological activities of essential oil, including antifungal antibacterial, used in pest and weed management were also discussed. Their use in the food industry as emulsion-based delivery systems, encapsulation, and edible coatings was discussed, highlighting its application in the postharvest preservation of fruits and vegetables. Due to the process constraints during essential oil manufacturing, economic, simple, and stable essential oil extraction techniques are in demand. Essential oil emulsions have been widely explored for their postharvest food preserving property. In a study, Galangal essential oil emulsion was prepared and tested for the quality maintenance of pineapple juice (Zhou et al.). Galangal essential emulsion at different concentrations was tested to influence the physical stability, physicochemical properties, microbial quantity, and aroma profiles of cloudy pineapple juice. It was found that the essential oil emulsion improved the physical stability of cloudy pineapple juice and the stability of the cloudy pineapple juice. Also, the microbial quantity of the cloudy pineapple juice was reduced by combining essential oil emulsion with thermal treatment. Galangal essential oil emulsion with quality indicators of cloudy pineapple juice suggested the potency of galangal essential oil as a juice preservative. Similarly, a novel cinnamaldehyde, eugenol, or carvacrol nanoemulsion was prepared and tested against *Penicillium digitatum*, the most severe pathogen that infects citrus fruits during storage (Yang et al.). It was found that these compound nanoemulsions had a striking inhibitory effect on *P. digitatum*. RNA-seq analysis revealed 2,169 differentially expressed genes between control and nanoemulsion-treated samples, including 1,028 downregulated and 1,141 upregulated genes. It was concluded that the antifungal activity of nanoemulsion was exerted due to multiple action sites against *P. digitatum*. This provides crucial information on developing novel formulations (site-specific) against the pathogenic microorganisms. Gum Arabic has been largely known for its wider applications in food, printing, paint production, glue, cosmetics, and various industrial applications, including viscosity control in inks and textile industries. Gum arabic edible coating was applied to reduce the postharvest decay and alleviate nutritional quality deterioration of ponkan fruit during cold storage (Huang et al.). It was found that the gum Arabic edible coating effectively reduces fruit decay and weight loss, retains higher total soluble solids content, suppresses titratable acidity degradation, and delays the ripening. It was recommended that gum arabic coating treatment used as a commercial wax to improve postharvest storability, extend storage life, and maintain the nutritional value of Ponkan fruit up to 120 days of cold storage. Another study reported that the exogenous γ-aminobutyric acid improves the postharvest quality of carambola fruit during low-temperature storage (Mekontso et al.). It was found that the γ-aminobutyric acid reduced the chilling injury index, maintained pericarp, decreased the electrolyte leakage and malondialdehyde content while increasing the superoxide dismutase, peroxidase, and catalase enzyme activities. Also, the chilling injury of tomato fruit was alleviated using Brassinolide (Bai et al.) and transcriptome, metabolome, and proteome analysis revealed the regulation mechanism of Brassinolide treatment in alleviating tomato fruit injury.

In conclusion, this special issue provides the current knowledge about the use and strategies of natural plant products for maintaining fruit quality and safety during postharvest storage. The use of natural products and advanced technologies provide a safer, effective, and economical material for the postharvest preservation of fruits and vegetables. However, these results after various trials need to be adopted by food industries to provide safer food products and thus reduce food security, hunger, and food losses. Besides these, the use of natural products in postharvest preservation would also limit the environmental damages and losses of the ecosystems compared to the synthetic chemicals and treatments.

## Author Contributions

CW wrote the introduction and the conclusion. KR wrote the central part with comments to the cited papers and references. All authors contributed to the article and approved the submitted version.

## Conflict of Interest

The authors declare that the research was conducted in the absence of any commercial or financial relationships that could be construed as a potential conflict of interest.

## Publisher's Note

All claims expressed in this article are solely those of the authors and do not necessarily represent those of their affiliated organizations, or those of the publisher, the editors and the reviewers. Any product that may be evaluated in this article, or claim that may be made by its manufacturer, is not guaranteed or endorsed by the publisher.
